# On the Security of a Simple Three-Party Key Exchange Protocol without Server's Public Keys

**DOI:** 10.1155/2014/479534

**Published:** 2014-09-01

**Authors:** Junghyun Nam, Kim-Kwang Raymond Choo, Minkyu Park, Juryon Paik, Dongho Won

**Affiliations:** ^1^Department of Computer Engineering, Konkuk University, 268 Chungwondaero, Chungju, Chungcheongbuk-do 380-701, Republic of Korea; ^2^Information Assurance Research Group, Advanced Computing Research Centre, University of South Australia, Mawson Lakes, SA 5095, Australia; ^3^Department of Computer Engineering, Sungkyunkwan University, 2066 Seobu-ro, Suwon, Gyeonggi-do 440-746, Republic of Korea

## Abstract

Authenticated key exchange protocols are of fundamental importance in securing communications and are now extensively deployed for use in various real-world network applications. In this work, we reveal major previously unpublished security vulnerabilities in the password-based authenticated three-party key exchange protocol according to Lee and Hwang (2010): (1) the Lee-Hwang protocol is susceptible to a man-in-the-middle attack and thus fails to achieve implicit key authentication; (2) the protocol cannot protect clients' passwords against an offline dictionary attack; and (3) the indistinguishability-based security of the protocol can be easily broken even in the presence of a passive adversary. We also propose an improved password-based authenticated three-party key exchange protocol that addresses the security vulnerabilities identified in the Lee-Hwang protocol.

## 1. Introduction

One of the fundamental problems in the areas of cryptography and communication security is to enable two parties communicating over a public network to establish a high-entropy secret key (known as a* session key*) from their low-entropy passwords which are easy for humans to remember. Password-based authenticated key exchange (PAKE) protocols are designed to solve this problem and often assume the three-party setting, in which each party (commonly called a client) needs to remember only a single password shared with a trusted server [1–11]. The design of secure yet efficient three-party PAKE protocols is notoriously hard and continues to be a subject of active research. A key challenge in designing such protocols is to prevent potential attacks by a malicious client, who is registered with the server, and thus is able to set up normal protocol sessions with other clients.

In this work, we present previously unpublished flaws in the S-EA-3PAKE protocol, a three-party PAKE protocol proposed by Lee and Hwang [4]. The design of the S-EA-3PAKE protocol is relatively simple and efficient and carries a claimed proof of security in the ROR model according to Abdalla et al. [1]. However, despite the claim of provable security, this protocol exhibits major security weaknesses. First, the protocol fails to achieve* implicit key authentication*, which is the fundamental security property that any given key exchange protocol is expected to provide. We demonstrate this by mounting a man-in-the-middle attack against the protocol. The attacker could be any malicious client. Second, the protocol is vulnerable to an offline dictionary attack by a malicious client and thus other clients cannot be guaranteed of the security of their passwords. Third, the protocol does not achieve semantic security of session keys; that is, session keys established by S-EA-3PAKE are distinguishable from random keys. We show this by mounting a passive attack in the ROR model, thereby invalidating the existing proof of security for S-EA-3PAKE. In addition to reporting the security vulnerabilities, we will also show how to fix the S-EA-3PAKE protocol so that it can achieve implicit key authentication as well as password security and semantic security.

Throughout the paper, we make the following assumptions on the capabilities of the adversary *A* in order to properly analyze the security properties of three-party PAKE protocols.

- *A* is either an outsider or an insider who runs in a probabilistic polynomial time.
- *A* has the complete control of all message exchanges between the server and clients. That is, *A* can eavesdrop, insert, modify, intercept, and delete messages exchanged among the protocol participants at will.

This assumption is the standard one [12, 13] and is consistent with Dolev-Yao model.

## 2. The S-EA-3PAKE Protocol

The S-EA-3PAKE protocol [4] is built upon Abdalla and Pointcheval's 2-party PAKE protocol called SPAKE [14]. Let *A* and *B* be two clients who wish to establish a session key, and *pw*_*A*_ and *pw*_*B*_ denote the passwords of *A* and *B*, respectively, shared with a trusted server *S*. The public parameters required by S-EA-3PAKE include

- a large prime *p* and a generator *g* of *Z*_p_*,
- two random elements *M* and *N* of *Z*_p_*,
- a cryptographic hash function *H* used as a key derivation function and
- a pair of message authentication code (MAC) generation/verification algorithms  (Mac,Ver), where  Ver outputs a bit, with 1 meaning accept and 0 meaning reject.

**Figure 1:**
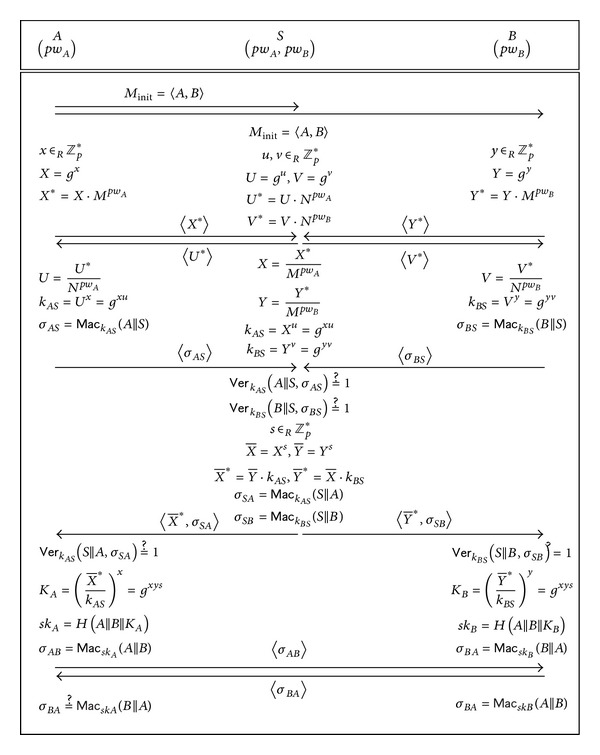
The S-EA-3PAKE protocol according to Lee and Hwang [4].

S-EA-3PAKE is shown in Figure 1 and proceeds as follows.

*Step 1*. *A* sends *S* and *B* a protocol initiation message *M*_init_ = 〈*A*, *B*〉 which states “*A* wants to establish a session key with *B*.”

*Step 2*. *A* and *S* establish a shared secret key *k*_*AS*_ by running the 2-party protocol SPAKE. Likewise, *B* and *S* establish a shared secret key *k*_*BS*_. More precisely, *k*_*AS*_ and *k*_*BS*_ are established as shown in Table 1.

*Step 3*. *A* (resp. *B*) computes the authenticator *σ*_*AS*_ = Mac_*k*_*AS*__(*A*||*S*) (resp. *σ*_*BS*_ = Mac_*k*_*BS*__(*B*||*S*)) and sends it to *S*.

*Step 4*. *S* aborts if either Ver_*k*_*AS*__(*A*||*S*, *σ*_*AS*_) = 1 or Ver_*k*_*BS*__(*B*||*S*, *σ*_*BS*_) = 1 is untrue. Otherwise, *S* selects a random *s* ∈ *Z*_p_*, computes

(1) X¯=Xs,Y¯=Ys,X¯∗=Y¯·kAS,Y¯∗=X¯·kBS,σSA=MackASsAsA,σSB=MackBSsB,

and sends $\langle {\bar{X}}^{*},\sigma_{SA}\rangle$ and $\langle {\bar{Y}}^{*},\sigma_{SB}\rangle$ to *A* and *B*, respectively.

*Step 5*. *A* checks if Ver_*k*_*AS*__(*S*||*A*, *σ*_*SA*_) = 1 and aborts if the check fails. Otherwise, *A* computes the key derivation secret, $K_{A}={\left({\bar{X}}^{*}/k_{AS}\right)}^{x}$, and the session key, *sk*_*A*_ = *H*(*A*||*B*||*K*_*A*_). Meanwhile, *B* checks if Ver_*k*_*BS*__(*S*||*B*, *σ*_*SB*_) = 1 and aborts if the check fails. Otherwise, *B* computes $K_{B}={\left({\bar{Y}}^{*}/k_{BS}\right)}^{y}$ and *sk*_*B*_ = *H*(*A*||*B*||*K*_*B*_).

*Step 6*. *A* and *B* perform key confirmation by exchanging *σ*_*AB*_ = Mac_*sk*_*A*__(*A*||*B*) and *σ*_*BA*_ = Mac_*sk*_*B*__(*B*||*A*) and verifying them in a straightforward way.

The correctness of S-EA-3PAKE can be easily verified from *K*_*A*_ = *K*_*B*_ = *g* ^*xys*^.

## 3. Previously Unpublished Flaws

### 3.1. No Implicit Key Authentication

Implicit key authentication of S-EA-3PAKE can be violated via a man-in-the-middle attack by a malicious (registered) client *C*. A possible attack scenario is as follows.

1. The attacker *C* blocks the protocol initiation message *M*_init_ = 〈*A*, *B*〉 from reaching *S* and instead sends (to *S*) two forged initiation messages *M*_init_′ = 〈*A*, *C*〉 and *M*_init_′′ = 〈*C*, *B*〉 which state, respectively, “*A* wants to establish a session key with *C*” and “*C* wants to establish a session key with *B*.” As a result, *S* will think that there are two protocol sessions running concurrently; let Π_*A*,*C*_ denote the session between *A* and *C* and let Π_*C*,*B*_ denote the session between *C* and *B*.
2. In both sessions Π_*A*,*C*_ and Π_*C*,*B*_, *C* performs Step 2 through 5 as per the protocol specification with its true identity. This can go undetected since none of the authenticators, *σ*_*AS*_, *σ*_*BS*_, *σ*_*SA*_, and *σ*_*SB*_, can confirm who the actual protocol participants are. As a result, *C* will share a session key, *sk*_*A*,*C*_, with *A* and another session key, *sk*_*C*,*B*_, with *B*.
3. With *sk*_*A*,*C*_ and *sk*_*C*,*B*_ in hand, *C* can perform Step 6 (of both sessions) in the straightforward way without being detected; *C* simply replaces *σ*_*AB*_ = Mac_*sk*_*A*,*C*__(*A*||*B*) and *σ*_*BA*_ = Mac_*sk*_*C*,*B*__(*B*||*A*) with *σ*_*AB*_′ = Mac_*sk*_*C*,*B*__(*A*||*B*) and *σ*_*BA*_′ = Mac_*sk*_*A*,*C*__(*B*||*A*), respectively.

At the end of the attack scenario, *A* and *B* believe that they have established a secure session with each other sharing a key, while in fact they have shared their keys with the attacker *C*. Consequently, S-EA-3PAKE fails to achieve implicit key authentication.

### 3.2. No Password Security

We now show that S-EA-3PAKE cannot protect clients' passwords against an offline dictionary attack. Assume a malicious client *C* who wants to find out the passwords of *A* and *B*. Let *pw*_*C*_ be the password of *C*. Then, an offline dictionary attack by *C* against both *A* and *B* can be mounted as follows.

**Phase 1 (gathering password verifiers online).**

*C* conducts a type of man-in-the-middle attack to obtain information required to verify password guesses.

1. *C* blocks the initiation message *M*_init_ = 〈*A*, *B*〉 from reaching *S* and instead sends two forged initiation messages *M*_init_′ = 〈*A*, *C*〉 and *M*_init_′′ = 〈*C*, *B*〉, thereby deceiving *S* into thinking that there are two protocol sessions, Π_*A*,*C*_ and Π_*C*,*B*_, running concurrently.
2. *C* then performs Step 2 through 5 of both sessions as specified by the protocol except for the following.
  - When *A* and *B* send X* = *X* · *M* ^*pw*_*A*_^ and Y* = *Y* · *M* ^*pw*_*B*_^ in Step 2, *C* makes a copy of these messages for later use.
  - *C* sends the same Step 2 message Z* = *g* ^*z*^ · *M* ^*pw*_*C*_^ of its own for both sessions, where *z*∈_*R*_ *Z*_p_*.
  - When *S* sends $\langle {\bar{X}}^{*},\sigma_{SA}\rangle$ and $\langle {\bar{Y}}^{*},\sigma_{SB}\rangle$, respectively, to *A* and *B* in Step 4 of the sessions, *C* replaces ${\bar{X}}^{*}$ and ${\bar{Y}}^{*}$ with ${\hat{X}}^{*}={\bar{X}}^{*}\cdot g^{z}$ and ${\hat{Y}}^{*}={\bar{Y}}^{*}\cdot g^{z}$, respectively.
3. Let $\langle {\bar{Z}}^{*},\sigma_{SC}\rangle$ and $\langle {\bar{Z^{\prime }}}^{*},\sigma_{SC}^{\prime }\rangle$ denote the two messages sent by *S* to *C* in Step 4 of Π_*A*,*C*_ and Π_*C*,*B*_, respectively.
4. Now when *A* and *B* exchange the key confirmation messages *σ*_*AB*_ = Mac_*sk*_*A*__(*A*||*B*) and *σ*_*BA*_ = Mac_*sk*_*B*__(*B*||*A*), *C* intercepts these messages and instead sends the clients “a failure message” to trick them into believing that, due to an unexpected error, their partner has failed to compute the session key and thus has aborted the protocol.

**Phase 2 (verifying password guesses offline).**

*C* can now verify password guesses both on *pw*_*A*_ and *pw*_*B*_ using the obtained information $\left(X^{*},{\bar{Z}}^{*},\sigma_{AB}\right)$ and $\left(Y^{*},{\bar{Z^{\prime }}}^{*},\sigma_{BA}\right)$, respectively. (For simplicity, we here describe this verification phase only for *pw*_*A*_; the case for *pw*_*B*_ proceeds correspondingly).

*Step 1*. *C* computes

(2) $$\begin{array}{c} K_{C}={\left(\frac{{\bar{Z}}^{*}}{k_{CS}}\right)}^{z}={\bar{X}}^{z}=g^{xzs}, \end{array}$$

where *k*_*CS*_ is the secret key shared between *C* and *S* in Step 2 of session Π_*A*,*C*_.

*Step 2*. Note that, since ${\bar{X}}^{*}$ was replaced with ${\hat{X}}^{*}={\bar{X}}^{*}\cdot g^{z}$, *A* must have computed *K*_*A*_ as

(3) KAX^∗kASx=X¯∗·gzkASx=gzs·gzx=gxzs·gxz.

With this in mind, *C* makes a guess *pw*_*A*_′ on the password *pw*_*A*_ and computes

(4) X′=X∗MpwA′,KA′=KC·X′z,skA′=HABKA′,σAB′=MacskA′AB.

*Step 3*. *A* verifies the correctness of *pw*_*A*_′ by checking that *σ*_*AB*_ is equal to *σ*_*AB*_′. If they are equal, then *pw*_*A*_′ is the correct password with an overwhelming probability.

*Step 4*. *C* repeats Steps 2  and  3 (of this verification phase) until a correct password is found.

This offline dictionary attack can be trivially simplified to an insider-attacker version whereby one of the two clients, *A* and *B*, tries to discover the other client's password. After all, the S-EA-3PAKE protocol cannot prevent any (malicious) client from mounting an offline dictionary attack against any other clients.

### 3.3. No Semantic Security

Finally, we point out that the S-EA-3PAKE protocol does not achieve the semantic security of session keys. In S-EA-3PAKE, the session key *sk*_*A*_ (resp. *sk*_*B*_) is used as the MAC key in generating the authenticator *σ*_*AB*_ = Mac_*sk*_*A*__(*A*||*B*) (resp. *σ*_*BA*_ = Mac_*sk*_*B*__(*B*||*A*)). This oversight leaks some information about the session key and allows an adversary to distinguish the real session key from a random key chosen from the session key space. Indeed, S-EA-3PAKE can be easily broken even in the presence of a passive adversary who asks only a single  Execute and  Test query. A simple attack by such an adversary *A* can be described as follows.

1. First, *A* makes an Execute(Π_A_*, Π_B_*, Π_S_*) query, where Π_A_*, Π_B_*, and Π_S_* denote any instance of *A*, *B*, and *S*, respectively. This query prompts an honest execution of the protocol between the three instances and will return the transcript of the protocol execution.
2. Next, *A* makes a Test(Π_A_*) query and receives a key $\bar{sk}$ in response to the query.
3. Then, *A* computes $\sigma_{AB}^{\prime }={\text{Mac}}_{\bar{sk}}(A||B)$ and checks if *σ*_*AB*_′ is equal to *σ*_*AB*_. The key $\bar{sk}$ is real if they are equal and otherwise it is random.

This attack invalidates the existing proof of security for S-EA-3PAKE [4]. We refer the reader to the work of Bellare et al. [13] for a possible countermeasure.

## 4. An Improved Three-Party PAKE Protocol

In this section, we propose an improved three-party PAKE protocol which achieves semantic security and is secure against man-in-the-middle attacks as well as offline dictionary attacks. Let *S* be the trusted server and let *A* and *B* be two registered clients of *S* who wish to establish a shared session key. We denote the passwords of *A* and *B* by *pw*_*A*_ and *pw*_*B*_, respectively. Our improved protocol uses the following public parameters:

- a finite cyclic group *G* of prime order *q* and a generator *g* of *G*;
- two random elements *M* and *N* of *G*;
- a cryptographic hash function *H* : {0,1}* → {0,1}^*l*^, where *l* represents the bit length of session keys;
- a pair of message authentication code (MAC) generation/verification algorithms (Mac, Ver), where Ver outputs a bit, with 1 meaning accept and 0 meaning reject.

**Figure 2:**
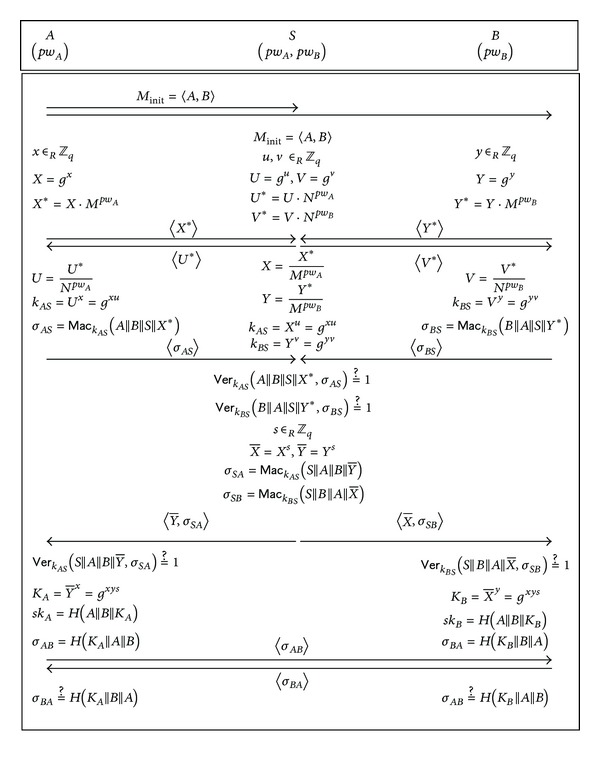
Our improved three-party PAKE protocol.

The improved protocol is illustrated in Figure 2 and its description is as follows.

*Step 1*. *A* sends *S* and *B* a protocol initiation message *M*_init_ = 〈*A*, *B*〉 which states “*A* wants to establish a session key with *B*.”

*Step 2*. *A* and *S* establish a shared secret key  *k*_*AS*_ by running the 2-party protocol SPAKE. Likewise, *B* and *S* establish a shared secret key *k*_*BS*_. More precisely, *k*_*AS*_ and *k*_*BS*_ are established as shown in Table 2. (Note in Table 2 that all the random exponents are selected from *Z*_*q*_ as our protocol works in a group of prime order *q*.)

*Step 3*. *A* (resp. *B*) computes the authenticator *σ*_*AS*_ = Mac_*k*_*AS*__(*A*||*B*||*S*||X*) (resp. *σ*_*BS*_ = Mac_*k*_*BS*__(*B*||*A*||*S*||Y*)) and sends it to *S*.

*Step 4*. *S* aborts if either Ver_*k*_*AS*__(*A*||*B*||*S*||X*, *σ*_*AS*_) = 1 or Ver_*k*_*BS*__(*B*||*A*||*S*||Y*, *σ*_*BS*_) = 1 is untrue. Otherwise, *S* selects a random *s* ∈ *Z*_*q*_, computes

(5) X¯=Xs,Y¯=Ys,σSA=MackASSABY¯,σSB=MackBSSABX¯,

and sends $\langle \bar{Y},\sigma_{SA}\rangle$ and $\langle \bar{X},\sigma_{SB}\rangle$ to *A* and *B*, respectively.

*Step 5*. *A* checks if ${\text{Ver}}_{k_{AS}}(S||A||B||\bar{Y},\sigma_{SA})=1$ and aborts if the check fails. Otherwise, *A* computes the key derivation secret, $K_{A}={\bar{Y}}^{x}$, and the session key, *sk*_*A*_ = *H*(*A*||*B*||*K*_*A*_). Meanwhile, *B* checks if ${\text{Ver}}_{k_{BS}}(S||B||A||\bar{X},\sigma_{SB})=1$ and aborts if the check fails. Otherwise, *B* computes $K_{B}={\bar{X}}^{y}$ and *sk*_*B*_ = *H*(*A*||*B*||*K*_*B*_).

*Step 6*. *A* and *B* perform key confirmation by exchanging *σ*_*AB*_ = *H*(*K*_*A*_||*A*||*B*) and *σ*_*BA*_ = *H*(*K*_*B*_||*B*||*A*) and verifying them in a straightforward way.

It can be easily verified that *A* and *B* compute session keys of the same value since *K*_*A*_ = *K*_*B*_ = *g* ^*xys*^. Compared with the S-EA-3PAKE protocol, our improved protocol does not require the computations of ${\bar{X}}^{*}$ and ${\bar{Y}}^{*}$ and simplifies the computations of *K*_*A*_ and *K*_*B*_. Therefore, it is fair to say that our protocol performs slightly better than the S-EA-3PAKE protocol.

Man-in-the-middle attacks and offline dictionary attacks such as the ones we mounted against the S-EA-3PAKE protocol are no longer valid against our improved protocol since the authenticators, *σ*_*AS*_, *σ*_*BS*_, *σ*_*SA*_, and *σ*_*SB*_, can now confirm who the actual protocol participants are. Moreover, our protocol achieves semantic security as the key derivation secrets *K*_*A*_ and *K*_*B*_ instead of the session keys *sk*_*A*_ and *sk*_*B*_ are used in generating the authenticators *σ*_*AB*_ = *H*(*K*_*A*_||*A*||*B*) and *σ*_*BA*_ = *H*(*K*_*B*_||*B*||*A*).

## 5. Concluding Remarks

The model where S-EA-3PAKE was claimed to be provably secure does not allow the adversary to ask Corrupt queries and thus cannot capture any kind of attacks that can be mounted by malicious clients. Accordingly, neither the man-in-the-middle attack nor the offline dictionary attack described in this work can be captured in the proof model. This situation is clearly unacceptable, from both theoretic and practical perspectives, and highlights the importance of considering Corrupt queries when proving security of three-party PAKE protocols. Although both the man-in-the-middle attack and the dictionary attack can be easily prevented by modifying the computations of the authenticators *σ*_*AS*_, *σ*_*BS*_, *σ*_*SA*_, and *σ*_*SB*_, the existence of a security proof for the S-EA-3PAKE protocol in a stronger model remains an open question. We finally note that all the three attacks presented in this work against S-EA-3PAKE also apply to the S-IA-3PAKE protocol [4], a simplified variant of S-EA-3PAKE. This becomes clear as soon as we notice that S-IA-3PAKE is different from S-EA-3PAKE only in the fact that it does not require the transmission of the authenticators *σ*_*AS*_,  *σ*_*BS*_, *σ*_*SA*_, and *σ*_*SB*_.

## Figures and Tables

**Table 1:** Establishing the secret keys *k*_*AS*_ and *k*_*BS*_ in the S-EA-3PAKE protocol.

*pw*_*A*_ → *k*_*AS*_	*pw*_*B*_ → *k*_*BS*_
*A* chooses a random *x* ∈ *Z*_p_*, computes *X* = *g* ^*x*^ and X* = *X* · *M* ^*pw*_*A*_^, and sends X* to *S*. At the same time, *S* chooses a random *u* ∈ *Z*_p_*, computes *U* = *g* ^*u*^ and U* = *U* · *N* ^*pw*_*A*_^, and sends U* to *A*. Then, *A* and *S* set *k*_*AS*_ = *g* ^*xu*^.	*B* chooses a random *y* ∈ *Z*_p_*, computes *Y* = *g* ^*y*^ and Y* = *Y* · *M* ^*pw*_*B*_^, and sends Y* to *S*. At the same time, *S* chooses a random *v* ∈ *Z*_p_*, computes *V* = *g* ^*v*^ and V* = *V* · *N* ^*pw*_*B*_^, and sends V* to *B*. Then, *B* and *S* set *k*_*BS*_ = *g* ^*yv*^.

**Table 2:** Establishing the secret keys *k*_*AS*_ and *k*_*BS*_ in our improved protocol.

*pw*_*A*_ → *k*_*AS*_	*pw*_*B*_ → *k*_*BS*_
*A* chooses a random *x* ∈ *Z*_*q*_, computes *X* = *g* ^*x*^ and X* = *X* · *M* ^*pw*_*A*_^, and sends X* to *S*. At the same time, *S* chooses a random *u* ∈ *Z*_*q*_, computes *U* = *g* ^*u*^ and U* = *U* · *N* ^*pw*_*A*_^, and sends U* to *A*. Then, *A* and *S* set *k*_*AS*_ = *g* ^*xu*^.	*B* chooses a random *y* ∈ *Z*_*q*_, computes *Y* = *g* ^*y*^ and Y* = *Y* · *M* ^*pw*_*B*_^, and sends Y* to *S*. At the same time, *S* chooses a random *v* ∈ *Z*_*q*_, computes *V* = *g* ^*v*^ and V* = *V* · *N* ^*pw*_*B*_^, and sends V* to *B*. Then, *B* and *S* set *k*_*BS*_ = *g* ^*yv*^.

## References

[1] Abdalla M, Fouque P, Pointcheval D (2005). Password-based authenticated key exchange in the three-party setting. *Public Key Cryptography-PKC 2005*.

[2] Nam J, Lee Y, Kim S, Won D (2007). Security weakness in a three-party pairing-based protocol for password authenticated key exchange. *Information Sciences*.

[3] Nam J, Paik J, Kang H, Kim UM, Won D (2009). An off-line dictionary attack on a simple three-party key exchange protocol. *IEEE Communications Letters*.

[4] Lee T, Hwang T (2010). Simple password-based three-party authenticated key exchange without server public keys. *Information Sciences*.

[5] Chang T, Hwang M, Yang W (2011). A communication-efficient three-party password authenticated key exchange protocol. *Information Sciences*.

[6] Zhao J, Gu D (2012). Provably secure three-party password-based authenticated key exchange protocol. *Information Sciences*.

[7] Wu S, Pu Q, Wang S, He D (2012). Cryptanalysis of a communication-efficient three-party password authenticated key exchange protocol. *Information Sciences*.

[8] Lee C, Chen S, Chen C (2012). A computation-efficient three-party encrypted key exchange protocol. *Applied Mathematics & Information Sciences*.

[9] Wu S, Chen K, Pu Q, Zhu Y (2013). Cryptanalysis and enhancements of efficient three-party password-based key exchange scheme. *International Journal of Communication Systems*.

[10] Nam J, Choo KKR, Kim M, Paik J, Won D (2013). Dictionary attacks against password-based authenticated three-party key exchange protocols. *KSII Transactions on Internet and Information Systems*.

[11] Nam J, Choo KKR, Kim J (2014). Password-only authenticated three-party key exchange with provable security in the standard model. *The Scientific World Journal*.

[12] Bellare M, Rogaway P (1994). Entity authentication and key distribution. *Advances in Cryptology—(CRYPTO '93)*.

[13] Bellare M, Pointcheval D, Rogaway P (2000). Authenticated key exchange secure against dictionary attacks. *Advances in Cryptology—EUROCRYPT 2000*.

[14] Abdalla M, Pointcheval D (2005). Simple password-based encrypted key exchange protocols. *Topics in Cryptology—CT-RSA 2005*.

